# Guideline adherence for early breast cancer before and after introduction of the sentinel node biopsy

**DOI:** 10.1038/sj.bjc.6602747

**Published:** 2005-08-23

**Authors:** M Schaapveld, E G E de Vries, R Otter, J de Vries, W V Dolsma, P H B Willemse

**Affiliations:** 1 Comprehensive Cancer Center North-Netherlands, PO Box 330, 9700 AH Groningen, The Netherlands; 2 Department of Medical Oncology, University Medical Center Groningen, PO Box 30.001, 9700 RB Groningen, The Netherlands; 3 Department of Surgical Oncology, University Medical Center Groningen, PO Box 30.001, 9700 RB Groningen, The Netherlands; 4 Department of Radiotherapy, University Medical Center Groningen, PO Box 30.001, 9700 RB Groningen, The Netherlands; 5 Department of Medical Oncology, University Medical Center Groningen, PO Box 30.001, 9700 RB Groningen, The Netherlands

**Keywords:** breast cancer, regional variation, breast-conserving surgery, guideline adherence, sentinel node biopsy

## Abstract

This population-based study aimed to analyse variations in surgical treatment and guideline compliance with respect to the application of radiotherapy and axillary lymph node dissection (ALND), for early breast cancer, before and after the sentinel node biopsy (SNB) introduction. The study included 13 532 consecutive surgically treated stage I–IIIA breast cancer patients diagnosed in 1989–2002. Hospitals showed large variation in breast-conserving surgery (BCS) rates, ranging between 27 and 72% for T1 and 14 and 42% for T2 tumours. In multivariate analysis marked inter-hospital and time-dependent variation in the BCS rate remained after correction for case-mix. The guideline adherence was markedly lower for elderly patients. In 25.2% of the patients aged ⩾75 years either ALND or radiotherapy were omitted. The proportion of patients with no ALND after an SNB increased from 1.8% in 1999 to 37.8% in 2002. However, in 2002 also 12.2% of the patients with a positive SNB did not have an ALND. Guideline compliance for BCS, with respect to radiotherapy and ALND, fell since the SNB introduction, from 96.1% before 2000 to 91.4% in 2002 (*P*<0.001). Noncompliance may however reflect patient-tailored medicine, as for elderly patients with small, radically resected primary tumours. The considerable variation in BCS-rates is more consistent with variations in surgeon preferences than patient's choice.

The establishment of a nationwide mammography breast-screening program for women aged 50–74 years has resulted in an increasing proportion of early breast cancers in the Netherlands during the 1990s (Nab *et al*, 1993; van Dijck *et al*, 2000). Over the last decade breast-conserving surgery (BCS) has become the standard treatment for early stage breast cancer, as firm data have shown that the outcome of BCS is comparable to the outcome after modified radical mastectomy (MRM) in terms of disease-specific survival (Veronesi *et al*, 1981; Fisher *et al*, 1985; EBCTG, 1995). Most breast cancer treatment guidelines state that the expected cosmetic outcome and patient preferences should guide the decision to perform BCS. Several studies, however, have indicated that surgical treatment for breast cancer may vary with the region or the hospital in which a patient is treated (Farrow *et al*, 1992; Nattinger *et al*, 1992; Voogd *et al*, 1994; Sainsbury *et al*, 1995; Scorpiglione *et al*, 1995; Bland *et al*, 1998; Guadagnoli *et al*, 1998b; Morrow *et al*, 2001). Although a large evidence base has been accumulated defining the most effective treatment strategies for early stage breast cancer, considerable treatment variability remains both between and within countries (Malin *et al*, 2002). Receiving less than appropriate care has been associated with an increased risk of recurrence and lower breast cancer-specific survival (Lash *et al*, 2000). Regular performance measurements, using standardised clinical indicators, can play an important role in monitoring the patterns of care with regard to cancer treatment (Schneider *et al*, 2004).

This population-based study presents an overview of treatment patterns for early stage breast cancer in the Netherlands over the period 1989–2002. The aim of this study was to evaluate the variation in primary surgical treatment and the compliance with guidelines, with respect to the application of radiotherapy and axillary lymph node dissection, with emphasis on the effect of the sentinel node biopsy (SNB) introduction.

## PATIENTS AND METHODS

### Patients

All surgically treated stage I–IIIA (excluding TNM stages T3N0–N2) breast cancer patients, diagnosed between January 1989 and January 2003 in the North-Netherlands and treated with either BCS or a MRM were eligible for inclusion. Patients with a prior invasive cancer, patients who received neo-adjuvant chemotherapy prior to surgery and patients with synchronous bilateral breast cancer, as defined by a contralateral breast cancer diagnosed within 3 months, were excluded.

### Breast-screening

A national breast-screening program, offering biennial mammography to women aged 50–69 years, was gradually implemented in the region since 1991. In 1997, all women in the target population had been invited at least once and since 1999 women aged 70–74 were also invited. All women received mammography in two directions for each breast: cranio-caudal and medio-latero-oblique. Two radiologists evaluated the mammograms by a double, independent reading. Women with a suspect mammogram were referred to the surgical department of one of the hospitals in the region for further evaluation.

### Data collection by the cancer registry

Data were collected by the regional cancer registry of the Comprehensive Cancer Center North (CCCN), covering the Northern Netherlands, a mainly rural area with a population of about 2.1 million, served by 16 community hospitals, one university medical centre, four radiotherapy facilities and seven pathology laboratories. PALGA, the nationwide Dutch network and registry of histo- and cytopathology, regularly submits reports of newly diagnosed malignancies to the cancer registry. The national hospital discharge databank, which receives discharge diagnoses of admitted patients from all Dutch hospitals, completes case ascertainment. After notification, trained registry personnel collect data on diagnosis, staging, and treatment from the medical records, including pathology and surgery reports. All primary treatment received is coded in sequence of administration. Patients are staged according to the TNM system of the UICC (Hermanek and Sobin, 1992; Sobin and Wittekind, 1997).

### Guidelines

The prevailing treatment guidelines for the study period are briefly outlined below. For patients with a tumour <4 cm, BCS with axillary lymph node dissection (ALND) was indicated, complemented with radiotherapy to the whole breast and a boost to the tumour excision area. The guidelines indicated that the surgical treatment should be based on the expected cosmetic outcome (tumour to breast ratio) and the patients' preferences. Alternatively, an MRM was performed. Loco-regional radiotherapy, consisting of parasternal, axillary, infra and supraclavicular nodal irradiation, was indicated in case of >3 positive axillary nodes or extranodal axillary growth. Parasternal irradiation was indicated for node-positive patients with a medially located tumour. Until 2000 ALND was indicated for all patients, after which a sufficient number of lymph nodes, at least 6 until 1998 and 10 thereafter, had to be pathologically examined. Since 2000 the guideline included the option of performing an SNB, for which a combined detection method was advised comprising peri-/intratumoral radioactive tracer injection and lymphoscintigraphy one day before surgery and blue dye injection at induction of anaesthesia. When a positive SNB was detected an ALND was indicated. Surgeons with sufficient, documented, experience with the SNB procedure (>30 SNB procedures with ALND as part of a learning curve) were allowed to omit ALND in patients with a negative SNB.

For the evaluation of guideline compliance, breast-conserving therapy was scored as ‘appropriate’ when it included an ALND and was complemented with radiotherapy; an MRM was considered in accordance with the guideline if complemented by an ALND. Omission of ALND was allowed after a negative SNB. An ALND with >3 positive nodes was considered an indication for regional radiotherapy, omission of radiotherapy was scored as ‘inappropriate’. Omission of radiotherapy for node-positive medially located tumours was also scored as ‘inappropriate’ in the evaluation of guideline adherence.

### Statistical analysis

The pathological tumour size was used to assess the choice of surgical treatment. In univariate analysis the *χ*^2^ test was used to examine the associations of categorical variables with the proportion of patients treated with BCS. The inter-hospital variation in the proportion of BCS was studied with Poisson regression analysis, adjusting for various patient and tumour characteristics. The rate of BCS was estimated against the regional average BCS rate as reference. Variables considered in the model were the hospital, patient age (<40, 40–49, 50–59, 60–69, 70–79 and 80+), tumour localisation in the breast (central/nipple, medial, lateral, overlapping), tumour size (⩽1 cm or T1a/1b, 1–2 cm or T1c, 2.1–5 cm or T2), year of diagnosis (1989–1991, 1992–1994, 1995–1997, 1998–2000, 2001–2002), mode of detection (screen-detected *vs* non-screen-detected) and distance from the nearest radiotherapy facility. Furthermore, first-order interactions of significant variables were tested (hospital with period of diagnosis, age at diagnosis and tumour size). Model fit was evaluated using the Pearson *χ*^2^ goodness-of-fit test statistic (McCullagh and Nelder, 1989). All reported *P*-values are two sided. A *P*-value <0.05 was considered significant.

## RESULTS

### Type of surgery

**Figure 1:**
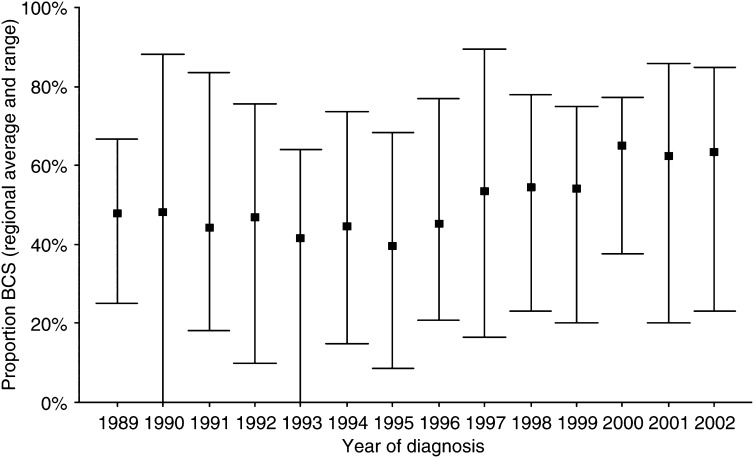
Inter-hospital variation in the proportion of T1 tumours treated with breast-conserving surgery (BCS) by year of diagnosis. The square represents the regional average; the bars represent the range between all hospitals.

**Figure 2:**
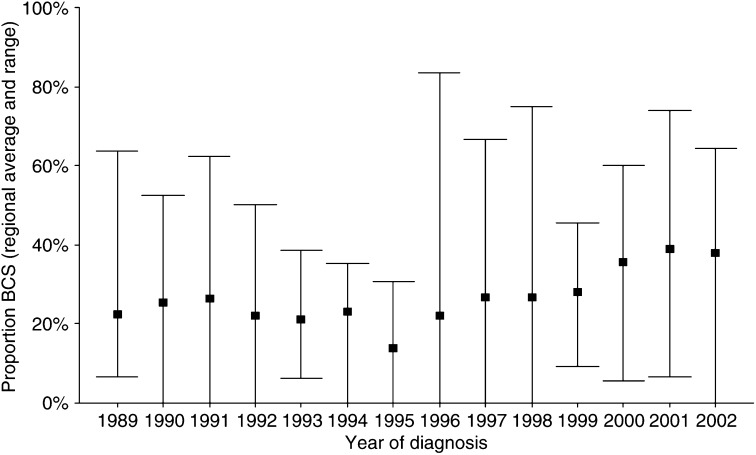
Inter-hospital variations in the proportion of T2 tumours treated with breast-conserving surgery (BCS) by year of diagnosis. The square represents the regional average; the bars represent the range between all hospitals.

Between January 1989 and January 2003, 13 532 consecutive patients were included. Table 1 shows some patient characteristics for patients receiving BCS. In total, 41.2% of the patients received BCS, 52.1% for a T1 and 26.5% for a T2 tumour. The proportion of patients treated with BCS varied markedly between the hospitals, ranging from 27.2 to 71.9% for T1 and from 13.5 to 42.3% for T2 tumours. Following a decrease between 1989 and 1995, the proportion receiving BCS gradually increased since 1996 for both T1 and T2 tumours (Figures 1 and 2). The initial decrease was most pronounced for patients <50 years, whereas for patients of 70+ years the BCS rate remained more or less stable until 1996.

Screen-detected tumours were better candidates for BCS. The screen-detected T1 tumours were smaller than non-screen-detected tumours. The proportion of tumours ⩽1 (T1A/B) and 1–2 cm (T1C) were 36.5 and 63.5% for screen-detected *vs* 20.6 and 79.4% for non-screen-detected tumours, respectively (*P*<0.001). Furthermore, since the completion of the implementation phase of the screening program for women aged 50–69 years, the proportion of nonpalpable screen-detected tumours increased from 44.2 in 1997 to 57.3% in 2002. Therefore, patients with screen-detected tumours actually were more likely to receive BCS (Table 2). Patients aged 50–69 and 70–74 years with a screen-detected T1 tumour had a, respectively, 1.15 (95% CI 1.08–1.22) and 1.81 (95% CI 1.55–2.10) fold higher relative risk (RR) of receiving BCS. For the T2 tumours these figures were 1.37 (95% CI 1.20–1.56) and 2.14 (95% CI 1.48–3.09), respectively. The rate of BCS decreased with older age, with the notable exception of screen-detected T1 tumours. Only 23.1% of the patients ⩾70 years underwent BCS, compared to 49.8% of patients <50 years. Over the age of 80 years, BCS was applied in 17% of the patients with a T1 and 8.7% with a T2 tumour.

The distance from the municipality, where the patient lived, to the nearest radiotherapy facility correlated negatively with the proportion of patients receiving BCS, although the distance rarely exceeded 80 km. The trend of a decreasing rate of BCS with increasing distance from a radiotherapy facility was seen for all age groups, with the exception of patients ⩾70 years with T2 tumours, and persisted over time. However, stratified by hospital, the association between distance from a radiotherapy facility and BCS disappeared, indicating that hospital could also explain the observed association.

### Multivariate analysis for type of surgery

**Figure 3:**
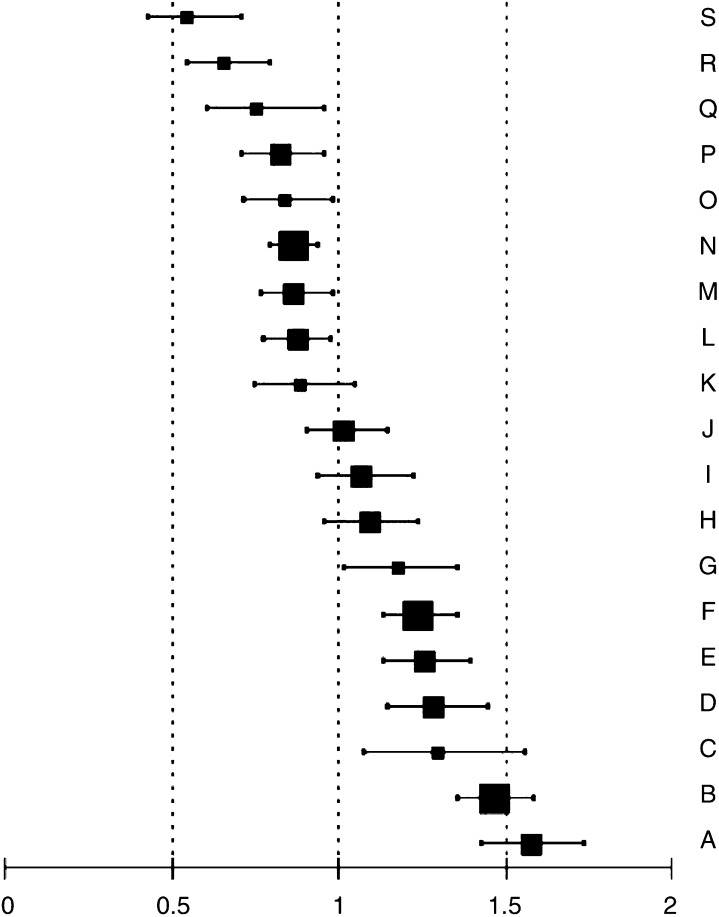
Estimated rate ratios with 95% confidence intervals for breast-conserving surgery (BCS) by hospital (denoted by the letters A–S) *vs* the regional average BCS rate (reference is 1.0) in the Comprehensive Cancer Center North region 1989–2002 (hospital with <500 patients 
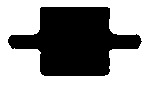
, with 500–999 patients 
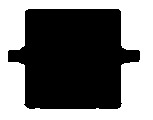
 and with ⩾1000 patients 
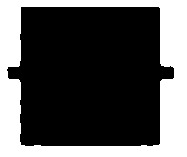
 diagnosed between 1989 and 2002).

In a Poisson regression analysis, older age, larger tumour size, lobular histology, central location or overlapping quadrants and a non-screen-detected cancer were all independently associated with a decreased BCS rate. Compared to patients with T1 tumours a patient with a T2 tumour had a RR of 0.58 (95% CI 0.54–0.63) of receiving BCS. Compared to patients <50 years, the RR of receiving BCS were 0.98 (95% CI 0.88–1.10), 0.87 (95% CI 0.77–0.97), 0.74 (95% CI 0.65–0.83), 0.52 (95% CI 0.46–0.60) and 0.26 (95% CI 0.21–0.32) for the 50–59, 60–69, 70–74, 74–79 and 80+ year age groups, respectively. A lobular histology decreased the rate of BCS by 26% (RR 0.74, 95% CI 0.66–0.82) and central tumour location or a tumour in overlapping quadrants decreased the rate of BCS with 18% (RR 0.82, 95% CI 0.75–0.89) and 31% (RR 0.69, 95% CI 0.60–0.80), respectively, compared to lateral or medial tumours. A non-screen-detected tumour was associated with a decreased BCS rate (RR 0.83, 95% CI 0.77–0.89). Even after correction for case mix, marked inter-hospital variation in the BCS rate remained. Following tumour size and patient age, the individual hospital was the most important variable predicting the likelihood of BCS (Figure 3). A strong effect of modification was found by year of diagnosis, the effect of time varying significantly between the hospitals (Table 3).

### Adjuvant radiotherapy after BCS

Of the 5577 patients who received BCS as definitive surgical therapy, 96.5% received radiotherapy. Withholding radiotherapy after BCS was associated with age. Whereas 97.7% of the patients <70 years received radiotherapy, these figures were 95.8, 90.9 and 57.4% for patients aged 70–74 years, 75–79 and ⩾80 years, respectively (*P*<0.001). Furthermore, the proportion of patients not receiving radiotherapy after BCS differed between the areas covered by the three regional radiotherapy facilities (5.0, 3.5 and 1.7%, respectively, *P*<0.001). Differences in treatment policy for the older patients partly explained this finding, as 63.2, 67.6 and 92.6% of the patients aged ⩾75 years received radiotherapy after BCS in the three radiotherapy facilities, respectively (*P*<0.001). Over time, the proportion of patients receiving radiotherapy following BCS remained stable, but the number of patients treated increased 2.7-fold between 1989 and 2002.

### Radiotherapy after MRM

Of the 7955 patients treated with an MRM, 21.6% subsequently received radiotherapy. Of these patients, 70.9% either had extensive lymph node involvement or a node-positive medial tumour. Of the patients who did not receive radiotherapy, 10.4% had these radiotherapy indications. Older patients were less likely to receive radiotherapy when indicated. Of the patients with a radiotherapy indication aged ⩾75 years, 52.9% actually received radiotherapy compared to 69.2% of the patients <75 years (*P*<0.001). Although the proportion of patients treated with radiotherapy differed slightly between the three radiotherapy facilities, after adjusting for radiotherapy indication this difference disappeared (*P*=0.254). Both the proportion and number of patients receiving radiotherapy after MRM remained stable over time.

### Axillary lymph node dissection and SNB

**Figure 4:**
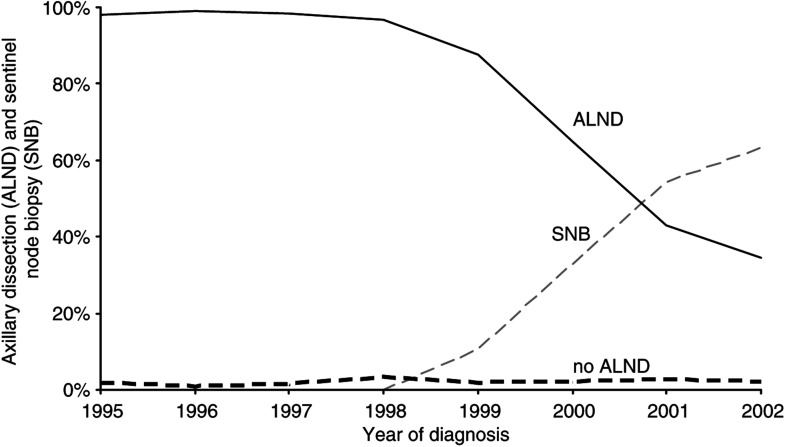
Time trend for sentinel lymph node biopsy and axillary lymph node dissection for breast cancer in the Comprehensive Cancer Center North region 1995–2002.

Before 2000, only 1.8% of the patients did not have an ALND (2.1% with a T1 and 1.4% with a T2 tumour). The proportion of patients without ALND increased from 1.0% in 1989 to 2.4% in 1998 and increased markedly with older age in this period, totalling 7.9% among patients aged ⩾80 years. The SNB was introduced at the end of 1998 and during 1998–2000 most patients received ALND after an SNB, as part of the surgeon's learning curve. Since 2000 the proportion of patients receiving an SNB increased. In 2002 only 34.8% of the patients still underwent an ALND without a prior SNB (Figure 4). The proportion of patients who underwent an SNB without a subsequent ALND increased steadily from 1.8% in 1999 to 37.8% in 2002. The proportion of patients without ALND after a positive SNB increased markedly in 2002 to 12.2%.

### Adherence to the guidelines for primary treatment

Table 4 illustrates the guideline adherence for early stage breast cancer. Following BCSn 95.0% of the patients underwent an ALND and subsequently received radiotherapy. The guideline adherence for breast-conserving therapy decreased following the SNB introduction, from 95.6% in 1999 to 91.3% in 2002 compared to, on average, 96.8% in the previous years (*P*<0.001). During 2001–2002, 3.1% of the patients treated with BCS did not have radiotherapy, 4.2% did not have ALND and in 1.3% both radiotherapy and ALND were omitted. Of the patients with no ALND, 54.8% had a positive SNB. The compliance with breast-conserving therapy guidelines decreased with older patient age (*P*<0.001). During 1989–2002, 25.2% of the patients aged ⩾75 years received ‘inappropriate’ breast-conserving therapy (omission of radiotherapy, ALND or both). Guideline compliance for breast-conserving therapy was lower following the SNB introduction in all age groups.

Guideline compliance for patients treated with an MRM averaged 90.6%, predominantly due to omission of radiotherapy for patients with (extensive) lymph node involvement. Of the 1642 patients with >3 positive (or fixed nodes/extracapsular tumour extension) axillary nodes, 39.4% did not receive radiotherapy although it was indicated. When indicated, radiotherapy was more frequently omitted in patients <50 years or ⩾75 years compared to patients aged 50–74 years. In 1.8% of the patients an ALND was incorrectly omitted; this proportion increased with older age and in the most recent years. The guideline compliance was lowest for patients aged ⩾75 years due to relatively frequent omission of either radiotherapy or ALND.

## DISCUSSION

In this population-based study, large inter-hospital variation in BCS was observed, which persisted after adjustment for case-mix. Following tumour size and age, the individual hospital was the most important variable predicting the likelihood of receiving BCS. Generally, hospitals, which scored far under or above the regional average, did so during the whole study period. The time trend for BCS varied significantly between the hospitals. Besides changes in doctors and patients attitudes towards BCS, changes in the surgical staff are a possible explanation for this observation. It is very likely that the observed inter-hospital variation in BCS reflects surgeon preference more than patient preference.

A study evaluating the effect of an interactive treatment decision aid in a Dutch patient population (*N*=172) showed that the patients' perception of her physicians' treatment preference was an important factor in decision making (Molenaar *et al*, 2004). In a population-based study, Katz *et al* found that patients who did not feel they had had a choice between surgical options perceived less satisfaction with the decision-making process (Katz *et al*, 2001). Informed decision making by the patient does not necessarily imply that a patient will choose BCS, however. A survey among 1489 patients in the Detroit and Los Angeles metropolitan area, performed shortly after surgery, found that patients who felt involved in the decision making were actually more inclined to accept MRM, whereas patients who underwent BCS felt more frequently that their surgeon made the treatment decision (Katz *et al*, 2004). In a study in Western Australia, women who received BCS also reported a more important role of the surgeon's preference in their decision-making than those who had had an MRM (Mastaglia and Kristjanson, 2001). A study, evaluating a decision board to help surgeons inform breast cancer patients about their treatment options, also had interesting effects. While the surgeons stated that the instrument improved communication and facilitated shared decision-making, the rate of BCS decreased after its introduction (Whelan *et al*, 1999). It remains therefore questionable whether the rate of BCS can be used indiscriminately as a standard for good quality of care. Nevertheless, several studies have shown that various quality of life indicators may differ between patients treated with BCS or MRM (Ganz *et al*, 1992; Pozo *et al*, 1992; Poulsen *et al*, 1997; Arora *et al*, 2001; Janni *et al*, 2001; Engel *et al*, 2004). Recently, it was shown that patients who underwent an MRM scored worse on body image, sexual functioning and lifestyle disruption compared to patient treated with BCS, while these scores did not improve over time in either patient group (Engel *et al*, 2004). Previous studies also reported that especially younger patients scored particularly worse on body image after an MRM than patients treated with BCS (Ganz *et al*, 1992; Arora *et al*, 2001).

The proportion of patients treated with BCS in our population was comparable to that in the USA, according to the data from the SEER registry (Lazovich *et al*, 1999). In the Southeast-Netherlands, 67% of stage I and 43% of stage II breast cancers received BCS in 1990–1991 (Voogd *et al*, 1994), proportions which were attained only in some hospitals in our region.

A temporary decrease in the BCS rate was observed during the mid-1990s. We can only speculate about the cause. The decrease manifested following publications showing an increased local recurrence risk after BCS for patients with larger tumours, tumours with an extensive *in situ* component and for patients younger than 40 years (Delouche *et al*, 1987; Bartelink *et al*, 1988; Boyages *et al*, 1990; Lichter *et al*, 1992). Also, in this period the breast-screening program rapidly expanded, which may have resulted in logistic problems in hospitals and radiotherapy facilities. The number of patients diagnosed with early stage breast cancer increased almost 40% in the period 1995–1997 compared to 1989–1991. On the other hand, in our study actually patients with screen-detected, early stage breast cancer were more likely to receive BCS, even after adjusting for tumour size. A study in the Southeast-Netherlands also found a higher likelihood of BCS for patients with screen-detected cancers, although this study did not correct for differences in tumour size between screen-detected and non-screen-detected cancers (Ernst *et al*, 2001).

Primary therapy generally was given in accordance with the guidelines. In all, 95% of the patients treated with BCS underwent ALND and received radiotherapy. Most patients (98.5%) had an ALND as part of an MRM. These results compare favourably with studies from the USA (Guadagnoli *et al*, 1998b; Lazovich *et al*, 1999; Nattinger *et al*, 2000; Morrow *et al*, 2001). Nattinger *et al* observed an increasing trend of inappropriate treatment of early stage breast cancer in the SEER database, mainly due to an increased proportion of patients receiving breast-conserving therapy and the higher likelihood of inappropriate breast-conserving therapy (omission of ALND, radiotherapy or both) compared to MRM; 19% of the patients treated in 1995 received incomplete treatment (Nattinger *et al*, 2000). In our population, the proportion of patients treated in accordance with the guideline fell since 1998, following the introduction of the SNB, frequently due to omission of ALND. Several studies have reported lower use of ALND and postoperative radiotherapy in the elderly patient (Voogd *et al*, 1994; Guadagnoli *et al*, 1998a; Hebert-Croteau *et al*, 1999; Edge *et al*, 2002; Giordano *et al*, 2005). The benefit of ALND for elderly patients has been seriously questioned in the literature (Wazer *et al*, 1994; Newlin *et al*, 2002; Martelli *et al*, 2003) and surgeons may be reluctant to perform an additional ALND (following BCS or SNB) in elderly patients as they frequently suffer from comorbidity. Over the years 2001–2002, in our study 50% of the patients who did not have an ALND had a tumour positive SNB; most of these patients were over 50 years of age. One could argue that the outcome of ALND in this group of patients would not often change the projected adjuvant treatment and as such may represent appropriate patient-tailored medical practice. In our population radiotherapy, as part of BCS, was omitted in 22% of the patients aged ⩾75 years. A recent CALGB-study, comparing lumpectomy plus tamoxifen with and without radiation in women with clinical stage I breast cancer aged ⩾70 years, found only a small nonsignificant excess risk of local recurrence in the nonirradiated group and no differences in distant metastases risk or survival (Hughes *et al*, 2004). Another recent study examined local recurrences rates among patients who refused radiotherapy or had medical contraindications and found low local recurrence rates among elderly patients with small, lower grade tumours operated with adequate resection margins (Lee *et al*, 2004). Although inappropriate according to the guideline, omitting radiotherapy after BCS in the very elderly appears to be reasonable medical practice for elderly patients with small, adequately resected tumours.

The prevailing guideline for elective nodal irradiation was largely based on the extent of nodal involvement during the study period. A relatively recent meta-analysis showed that postoperative locoregional radiotherapy resulted in a survival advantage for high-risk patients (Whelan *et al*, 2000). Other studies have shown that even after an adequate axillary dissection and adjuvant systemic therapy, a high risk of locoregional recurrence remained in patients with a high number of involved nodes when these patients did not receive postoperative radiotherapy (Ragaz *et al*, 1997; Recht *et al*, 1999). Although it is as yet not completely clear which patients do need locoregional radiotherapy, in the Netherlands the current guidelines advise to give axillary and supraclavicular radiotherapy in case of >3 positive axillary lymph nodes or a positive apical node. In our study, 39% of the patients treated with an MRM for whom radiotherapy was indicated according to this guideline actually were not irradiated. Comorbidity and older age have previously been associated with decreased use of loco-regional radiotherapy (Ballard-Barbash *et al*, 1996; Hebert-Croteau *et al*, 1999; Morrow *et al*, 2001). There is a need for guidelines, which better address treatment issues in the elderly breast cancer patient, especially as the elderly comprise a growing proportion of our patient populations.

This study provides an evaluation of current treatment patterns for breast cancer. To ensure that these results would improve the quality of care, the data were presented to delegations from all regional hospitals, including delegates from the surgical staff, during three invitational conferences in the first half of 2005. Unit names were not removed in these presentations. The variation in BCS has been discussed repeatedly within the Comprehensive Cancer Center North (CCCN) breast cancer working group and more recently within the Surgical Oncological Network North Netherlands, a CCCN working group comprising surgeons from all hospitals in the CCCN region. As a result of these discussions, the CCCN cancer registry provides since 2003, among other data, stage and age specific rates of BCS for each hospital in the CCCN region. These data are regularly discussed within the Surgical Oncological Network North Netherlands, during which the results of individual units are opened to all other hospitals and are compared to the regional average and that of other centres. Largely as a result of these discussions we have seen a marked increase in the proportion of patients treated with BCS.

## Figures and Tables

**Table 1:** Characteristics of all patients and those receiving breast-conserving surgery (BCS)

	**All patients**	**Patients receiving BCS**	
	**Number**	**Number**	**%**
*Tumour location*			
Central	878	239	27.2
Medial	2788	1260	45.2
Lateral	6708	2950	44.0
Overlapping	3158	1128	35.7
			
*Tumour size*			
T1A/B	2023	1178	58.2
T1C	5766	2878	49.9
T2	5743	1521	26.5
			
*Age (years)*			
<50	3473	1718	49.5
50–69	6645	3079	46.3
70–74	1450	478	33.0
75+	1964	302	15.4
			
*Total number of patients treated per hospital*			
Average	712	293	41.2
Range	222–2231	63–793	21.5–60.8
			
*Distance from radiotherapy department (km)*			
0–9.9	2439	1111	45.6
10–24.9	2681	1149	42.9
25–44.9	5470	2266	41.4
45+	2896	1025	35.4
			
*Year of diagnosis*			
1989–1991	2145	769	35.9
1992–1994	2667	897	33.6
1995–1997	2951	1049	35.5
1998–2000	3265	1531	46.9
2001–2002	2504	1331	53.2
			
*Mode of detection*			
Screen detected	3327	1830	55.0
Non-screen detected	10 205	3747	36.7
			
*Histology*			
Ductal carcinoma	11 484	4943	43.0
Lobular carcinoma^a^	1429	430	30.1
Other^b^	617	203	32.9

a Including mixed lobular and ductal carcinoma.

b Mucinous, medullary, unspecified.

**Table 2:** Breast-conserving surgery (BCS) rate according to the mode of detection, age and tumour size

		**T1**		**T2**	
		**BCS**	**Total**	**BCS**	**Total**
**Mode of detection**	**Age (years)**	**%**	***N***	**%**	***N***
Non-screen detected	<50	60.4	1876	36.0	1535
	50–69	52.0	1969	27.5	1874
	70–74	33.8	488	14.9	545
	75+	19.5	826	10.5	1092
	Total	48.1	5159	25.0	5046
					
Screen detected	<50	58.0	50	25.0	12
	50–69	59.5	2210	38.0	592
	70–74	61.2	335	32.9	82
	75+	68.6	35	18.2	11
	Total	59.8	2630	36.9	697

**Table 3:** Results of multivariate Poisson regression analysis for variation in the rate of breast-conserving surgery (BCS) and estimated rate ratios (RR) of BCS by hospital for each period of diagnosis (with 1989–1991 as reference)

			**Time trend for BCS**				
			**1989–1991**	**1992–1994**	**1995–1997**	**1998–2000**	**2001–2002**
	**RR^a^**	**95% CI**	**RR**	**RR**	**RR**	**RR**	**RR**
*Hospital*							
Hospital A	1.89	1.14–3.12	1.00	0.72	0.83	0.99	1.14
Hospital B	1.87	1.18–2.95	1.00	0.85	0.84	0.97	0.98
Hospital C	1.93	1.06–3.50	1.00	0.40	0.88	0.92	1.01
Hospital D	1.38	0.80–2.37	1.00	1.09	0.98	1.11	1.51
Hospital E	1.68	1.02–2.76	1.00	0.83	0.81	0.78	0.95
Hospital F	1.30	0.79–2.12	1.00	0.97	1.03	1.22	1.43
Hospital G	1.66	0.95–2.89	1.00	0.78	1.02	0.62	0.74
Hospital H	0.96	0.63–1.46	1.00	1.15	0.95	1.64	1.58
Hospital I	0.91	0.51–1.61	1.00	0.92	0.95	1.69	1.88
Hospital J	1.04	0.61–1.75	1.00	0.81	0.73	1.39	1.66
Hospital K	1.26	0.68–2.31	1.00	0.77	0.77	0.85	0.87
Hospital L	0.61	0.34–1.10	1.00	1.63	1.55	1.86	2.30
Hospital M	0.71	0.37–1.36	1.00	1.05	0.81	1.45	2.54
Hospital N	0.98	0.61–1.56	1.00	0.79	0.67	1.36	1.38
Hospital O	0.53	0.25–1.14	1.00	1.10	1.81	2.33	2.94
Hospital P	1.03	0.57–1.86	1.00	1.00	0.94	1.31	0.58
Hospital Q	1.63	0.80–3.32	1.00	0.52	0.52	0.56	0.71
Hospital R	0.61	0.31–1.17	1.00	1.23	0.88	2.19	1.46
Hospital S	0.58	0.26–1.27	1.00	1.00	1.02	1.38	1.06

a Relative risk of BCS for the period 1989–1991 *vs* the regional average BCS rate, adjusted for age, tumour size, period of diagnosis, histology, location and mode of detection.

95% CI: 95% confidence interval for RR.

**Table 4:** Guideline adherence for axillary lymph node dissection (ALND) and radiotherapy (RT) in breast cancer treatment, by type of surgery, age and period of diagnosis

	**Therapy not according to guideline**						**Therapy according to guideline**		
	**No ALND**		**No RT**		**No RT and no ALND**				**Total**
**Type of surgery**	***N***	**%**	***N***	**%**	***N***	**%**	***N***	**%**	***N***
*BCS*									
Total	88	1.6	133	2.4	60	1.1	5296	95.0	5577
									
*Age (years)*									
<50	14	0.8	38	2.2	1	0.1	1665	96.9	1718
50–69	60	1.9	55	1.8	12	0.4	2952	95.9	3079
70–74	4	0.8	14	2.9	7	1.5	453	94.8	478
75+	10	3.3	26	8.6	40	13.2	226	74.8	302
									
*Year of diagnosis*									
1989–1991	1	0.1	23	3.0	—	—	745	96.9	769
1992–1994	1	0.1	30	3.3	3	0.3	863	96.2	897
1995–1997	8	0.8	6	0.6	8	0.8	1027	97.9	1049
1998–2000	22	1.4	32	2.1	32	2.1	1445	94.4	1531
2001–2002	56	4.2	42	3.2	17	1.3	1216	91.4	1331
									
*MRM*									
Total	116	1.5	628	7.9	2	0.0	7209	90.6	7955
									
*Age (years)*									
<50	12	0.7	185	10.5	—	—	1558	88.8	1755
50–69	17	0.5	229	6.4	1	0.0	3319	93.1	3566
70–74	9	0.9	60	6.2	—	—	903	92.9	972
75+	78	4.7	154	9.3	1	0.1	1429	86.0	1662
									
*Year of diagnosis*									
1989–1991	23	1.7	74	5.4	—	—	1279	93.0	1376
1992–1994	19	1.1	158	8.9	—	—	1593	90.0	1770
1995–1997	18	0.9	178	9.4	—	—	1706	89.7	1902
1998–2000	28	1.6	120	6.9	—	—	1586	91.5	1734
2001–2002	28	2.4	98	8.4	2	0.2	1045	89.1	1173
